# Dose-Dependent Effects of L-Arginine on PROP Bitterness Intensity and Latency and Characteristics of the Chemical Interaction between PROP and L-Arginine

**DOI:** 10.1371/journal.pone.0131104

**Published:** 2015-06-23

**Authors:** Melania Melis, Massimiliano Arca, Maria Carla Aragoni, Tiziana Cabras, Claudia Caltagirone, Massimo Castagnola, Roberto Crnjar, Irene Messana, Beverly J. Tepper, Iole Tomassini Barbarossa

**Affiliations:** 1 Department of Biomedical Sciences, Section of Physiology, University of Cagliari, Monserrato, CA, Italy; 2 Dipartimento di Scienze Chimiche e Geologiche, Università degli Studi di Cagliari, Monserrato, CA, Italy; 3 Department of Life and Environment Sciences, Macrosection of Biomedicine, University of Cagliari, Monserrato, CA, Italy; 4 Institute of Biochemistry and Clinical Biochemistry, Catholic University, Rome, Italy; 5 Department of Food Science, School of Environmental and Biological Sciences, Rutgers University, New Brunswick, New Jersey, United States of America; The University of Tokyo, JAPAN

## Abstract

Genetic variation in the ability to taste the bitterness of 6-n-propylthiouracil (PROP) is a complex trait that has been used to predict food preferences and eating habits. PROP tasting is primarily controlled by polymorphisms in the *TAS2R38* gene. However, a variety of factors are known to modify the phenotype. Principle among them is the salivary protein Ps-1 belonging to the basic proline-rich protein family (bPRP). Recently, we showed that oral supplementation with Ps-1 as well as its related free amino acids (L-Arg and L-Lys) enhances PROP bitterness perception, especially for PROP non-tasters who have low salivary levels of Ps-1. Here, we show that salivary L-Arg levels are higher in PROP super-tasters compared to medium tasters and non-tasters, and that oral supplementation with free L-Arg enhances PROP bitterness intensity as well as reduces bitterness latency in a dose-dependent manner, particularly in individuals with low salivary levels of both free L-Arg and Ps-1 protein. Supplementation with L-Arg also enhanced the bitterness of caffeine. We also used ^1^H-NMR spectroscopy and quantum-mechanical calculations carried out by Density Functional Theory (DFT) to characterize the chemical interaction between free L-Arg and the PROP molecule. Results showed that the –NH_2_ terminal group of the L-ArgH^+ ^side chain interacts with the carbonyl or thiocarbonyl groups of PROP by forming two hydrogen bonds with the resulting charged adduct. The formation of this PROP•ArgH+ hydrogen-bonded adduct could enhance bitterness intensity by increasing the solubility of PROP in saliva and its availability to receptor sites. Our data suggest that L-Arg could act as a ‘carrier’ of various bitter molecules in saliva.

## Introduction

The sense of taste guides organisms to recognize nutrient-rich food from noxious substances, and is the final arbiter that controls food acceptance or rejection behaviors [1, 2]. Taste perception varies greatly across individuals and, by influencing food preferences, may have important consequences for nutritional status and health [3]. Since many bitter-tasting substances can be toxic, the ability of humans to detect bitterness at low concentrations can represent an evolutionary adaptation for limiting the consumption of these substances [3]. On the other hand, several classes of bitter polyphenols found in tea, coffee, and chocolate, provide positive health benefits, so low sensitivity encourages their consumption [4]. Within this broad nutritional context, variation in sensitivity to the bitter taste of 6-n-propylthiouracil (PROP) has been widely studied since it associates with the perception of a wide range of oral stimuli, food preferences, dietary behavior and nutritional status [3, 5]. It has been shown that subjects who detect PROP only at high concentrations, or not at all, are defined non-tasters. Non-tasters show reduced sensitivity to oral fat that is associated with increased preference and dietary intake of high-fat/high-energy foods [6–10]. On the contrary, subjects who are moderately or very sensitive to PROP (medium tasters and super-tasters), show greater sensitivity to oral fat and lower liking and intake of high-fat and strong-tasting food [3, 11, 12]. These findings support the hypothesis of an inverse correlation between PROP tasting and food intake, calorie consumption and BMI, which has been reported in several studies [9, 13–16].

The genetic ability to taste PROP is strongly associated with haplotypes of the bitter receptor TAS2R38, which can explain most phenotypic differences in PROP tasting [17, 18]. Allelic diversity in *TAS2R38* gives rise to two common haplotypes: PAV, the dominant variant with high affinity for the N—C = S group, which is responsible for the bitter taste of PROP; and AVI, the non-taster recessive one. Rare haplotypes (AAI, AAV, and PVI) have been observed to contribute to intermediate sensitivity. PROP responsiveness has also been associated with a polymorphism (*rs2274333*) located in the gustin (CA6) gene that controls the zinc-dependent salivary protein of the same name [19]. Gustin protein had been described in the literature as a taste bud trophic factor [20]. Recently, Melis and coauthors [21] showed that this same polymorphism in the gustin gene affects PROP sensitivity by acting on cell growth and fungiform papillae maintenance. Subsequently, Barbarossa et al [22] reported that this polymorphism associates strongly with fungiform papilla density, whereas PROP bitterness associates with *TAS2R38* polymorphisms. Other studies failed to find associations between PROP tasting and gustin genotypes [23, 24].

A multiplicity of genetic and environmental factors have been shown to influence PROP perception [25]. Among them, the chemical composition of saliva, the health and integrity of taste cells [19, 26–28], and the number and morphology of fungiform taste papillae [21, 29–34] have been proven. Greater PROP responsiveness has been also related to high salivary levels of two specific proteins (Ps-1 and II-2) belonging to the basic proline-rich protein family (bPRP) [35, 36]. Recently, we showed that oral supplementation of these peptides facilitates PROP perception mostly in non-taster subjects [36]. We also observed a similar effect with supplementation with the free amino acid L-Arginine (L-Arg), which is highly represented in the sequence of Ps-1 and II-2 proteins and chemically interacts with the PROP molecule as demonstrated by ^1^H-NMR spectroscopy [36].

The primary objectives of this study were to investigate the physiological mechanisms by which L-Arg facilitates the perception of PROP, the relationship between PROP bitter taste responsiveness and basal levels of salivary free L-Arg, as well as the effect of supplementation with increasing concentrations of L-Arg on PROP bitter taste responsiveness. Subjects were phenotyped for PROP tasting and genotyped for *TAS2R38* and gustin gene polymorphisms. We used ^1^H-NMR spectroscopy to describe and examine the strength of the chemical interaction between L-Arg and PROP. The nature of this interaction was investigated using quantum-mechanical calculations carried out by means of the Density Functional Theory (DFT). An additional objective was to examine the effect of L-Arg supplementation on modulating the bitterness of caffeine, which is commonly consumed in the diet in coffee, tea and chocolate. The purpose of studying a second bitter molecule was to determine if the effect of L-Arg was specific to PROP or represented a more general mechanism of bitterness enhancement.

## Materials and Methods

### Ethics statement

Subjects were verbally informed about the procedure and aim of the study. All reviewed and signed an informed consent form. The Ethical Committee of the University Hospital of Cagliari approved the study procedures that have been performed in accordance with the latest revision of the Declaration of Helsinki.

### Subjects

Fifty-one non-smoking healthy white volunteers (14 men and 37 women) were recruited through public advertisements at the University of Cagliari. They ranged in age from 20 to 35 years (mean age 27.6 y ± 1.2 y) and they had a body mass index (BMI) ranging from 18.6 to 25.3 kg/m^2^. None were dieting or taking medications that might interfere with taste function. No variations in body weight larger than 5 kg were recorded over the 3 months preceding the enrollment. None of the subjects had food allergies, or scored high on eating behaviour scales (assessed by the Three-Factor Eating Questionnaire) [37]. In order to rule out any gustatory impairment, all participants tasted supra-threshold solutions of the 4 basic tastes (sweet, sour, salty, bitter).

### PROP taster status

In order to classify each subject for his/her PROP taster status, taste intensity ratings were collected using the 3-solution test [38, 39]. The test consists of three suprathreshold PROP (Sigma-Aldrich, Milan, Italy) (0.032, 0.32, and 3.2 mM) and sodium chloride (NaCl, Sigma-Aldrich, Milan, Italy) (0.01, 0.1, 1.0 M) solutions prepared with spring water. Solutions were prepared the day before each session and stored in the refrigerator until 1 h before testing. Stimuli were presented at room temperature.

Subjects were tested in two sessions separated by a 1-month period. They were requested to abstain from eating, drinking and using oral care products or chewing gums for at least 8 h prior to test session. In women, the taste assessments and saliva collection (described below) were done on the sixth day of the menstrual cycle to avoid taste sensitivity changes or fluctuations of levels of salivary components due to the oestrogen phase [40–43]. All subjects had to be in the test room 15 min before the beginning of the trials (at 9.30 AM) in order to adapt to the environmental conditions (23–24°C; 40–50% relative humidity) which were kept constant throughout the experimental sessions. The presentation order of the two taste stimuli (PROP or NaCl10 mL) was reversed in the two sessionsand concentrations were tasted in a random order within each solution type. Each stimulation was followed by oral rinsing with spring water. The interstimulus interval was set to 60 s. The taste intensity rating for each PROP or NaCl solution was recorded using the Labeled Magnitude Scale (LMS) [44] in which each subject placed a mark on the scale corresponding to his/her perception of the stimulus. The LMS scale gives subjects the freedom to rate the intensity of a stimulus relative to the “strongest imaginable” oral stimulus they have ever experienced in their life. The mean of ratings in the two replicates was calculated and perceived taste intensity functions for PROP and NaCl for each subject were generated from the results [13, 38]. 37.27% of the subjects were classified as non-tasters (n = 19), as they gave lower intensity ratings at the two highest concentrations of PROP as compared to the two highest concentrations of NaCl; 39.22% were classified as medium tasters (n = 20), as they gave similar ratings for PROP and NaCl at all concentrations; and 23.53% gave higher ratings to 0.32 and 3.2 mmol/l PROP as compared to the two highest concentrations of NaCl and were classified as super-tasters (n = 12). Three-way ANOVA was used to document the presence of the three taster groups (see S1 Table).

### Salivary analyses

#### Saliva collection and treatment

In the first visit, before starting taste assessments, two samples (1 mL) of whole unstimulated saliva were collected from each subject for L-Arg and Ps-1 quantitative determinations. Samples (1 mL) were collected for 1 min with a soft plastic aspirator as saliva flowed into the anterior floor of the mouth, and then transferred to a plastic tube. For Ps-1 analysis, each sample was immediately mixed with an equal volume of aqueous trifluoroacetic acid (0.2%); for L-Arginine determination each sample was immediately mixed with a half volume of aqueous trifluoroacetic acid (0.2%). Both samples were kept in an ice bath, in order to preserve and stabilize them by inhibiting salivary proteases. Samples were then centrifuged at 8000 g, at 4°C for 15 min. The acidic supernatant was separated from the precipitate and then immediately stored at -80°C until the HPLC-ESI-IT-MS analysis.

#### HPLC-ESI-IT-MS analysis

High performance liquid chromatography-electrospray ionization-ion trap-MS (HPLC-ESI-IT-MS) measurements were carried out by a Surveyor HPLC system connected by a T splitter to a diode-array detector and to an LCQ Advantage mass spectrometer equipped with an ESI source (ThermoFisher Scientific San Jose, CA). In order to quantify Ps-1 protein, saliva samples analysis was performed with a Vydac C8 chromatographic column (Grace, Hesperia, CA) with 5 μm particle diameter (150 x 2.1 mm) and by using chromatographic conditions previously reported [35, 36]. 100 μL of the acidic soluble fraction corresponding to 50 μL of whole unstimulated saliva was analysed by RP-HPLC-ESI-MS. The quantification of the Ps-1 protein was based on the area of the extracted ion current (XIC) peak. The XIC analysis reveals the peak associated with the protein of interest by searching along the total ion current chromatographic profile the specific multiply-charged ions generated at the source by the protein. The area of the ion current peak is proportional to concentration, and under constant analytical conditions it may be used to perform relative quantification of the same analyte in different samples [45, 46].

L-Arg was analysed by using an Alltima HP HILIC column (Grace, Hesperia, CA) with 5 μm particle diameter (250 x 2.1 mm). The following solutions were utilized for HPLC-ESI-MS analysis: (eluent A) aqueous 0.025% TFA and 0.5% acetic acid (v/v), (eluent B) acetonitrile containing 0.025% (v/v) TFA and 0.5% acetic acid. An isocratic elution was applied with 85% of eluent B for 6 minutes accordingly to Shin et al. [47] at a flow rate of 0.30 mL/min. The T splitter delivered a flow-rate of 0.20 ml/min toward the diode array detector and 0.10 ml/min toward the ESI source. The photodiode array detector was set at 214 and 276 nm. Mass spectra were collected every 3 ms in the positive ion mode. The MS spray voltage was 5.0 kV, and the capillary temperature was 235°C. MS spectra were collected in the range 50-220m/z. 100 μL of sample was injected into the HPLC-MS apparatus, corresponding to 67 μL of saliva.

Identification of L-Arg in salivary samples was performed in the Selected Reaction Monitoring (SRM) mode. The precursor mono-charged ion at 175.2 ± 0.1 m/z was isolated, fragmented (isolation width 2.0, 35% collision), and the three product ions at 60.1, 116.1 and 158.1 ± 0.1 m/z were detected. The peak area of the ion 60.1± 0.1 m/z was used for L-Arg quantification.

### Molecular analysis

Subjects were genotyped for three single nucleotide polymorphisms (SNPs) at base pairs 145 (C/G), 785 (C/T), and 886 (G/A) of the *TAS2R38* locus. The *TAS2R38* SNPs give rise to 3 non-synonymous coding exchanges: proline to alanine at residue 49, alanine to valine at residue 262 and valine to isoleucine at residue 296. Subjects were also genotyped for the gustin (CA6) gene polymorphism *rs2274333* (A/G) that correspond to the substitution Ser90Gly.

Molecular analyses were performed using PCR techniques followed by sequencing of the fragments obtained according to our previous works [19, 21, 28].

### ^1^H-NMR Spectroscopy-PROP/L-Arg interaction

The interaction between PROP and different amounts of free L-Arg was investigated by ^1^H-NMR spectroscopy. This technique permits the identification and evaluation of the chemical interaction of proteins and/or specific amino acids with other compounds such as tannins, polyphenols or PROP [36, 48]. In fact, when a proton is involved in a noncovalent binding interaction it undergoes a field-shift (i.e., a modification in its chemical surrounding) resulting in a variation in the corresponding ^1^H-NMR signal. Recently, we used ^1^H-NMR spectroscopy to chemically probe the interaction between PROP and the free amino acids present in the Ps-1 and II-2 sequences [36], identifying L-Arg to be involved in the local binding of these peptides to the PROP molecule.

In order to evaluate how different L-Arg concentrations could affect the ^1^H-NMR signal previously attributed to PROP molecule, we recorded the ^1^H-NMR spectra of a PROP solution without L-Arg and after several additions of the amino acid up to a 1:1.625 PROP:L-Arg molar ratio. All experiments were recorded at 300 K using a Varian Inova 500 MHz FT-NMR system.

Spectra were processed and displayed using the MestReNova program. A solution of PROP (1.5 mL) in D_2_O (0.005 M) was prepared. Then, 0.5 mL of the PROP solution was placed in a 5 mL NMR tube, and the remaining 1 mL was used to prepare a L-Arg solution (0.034 M) in D_2_O. The experiments were performed by adding aliquots of the L-Arg solution to the solution of PROP. The ^1^H-NMR chemical shift change for the PROP ring proton in the absence and in the presence of different amounts of L-Arg was determined in terms of Δ = (|(δ'-δ_0_)|/δ_0_)·100, which represents the absolute value of the difference between the ^1^H-NMR signal (ppm) of the PROP ring proton in the absence (δ_0_) and in the presence (δ') of the amino acid, normalized for δ_0_ and expressed as a percentage.

### QM Computational investigation on PROP/L-Arg interaction

Computational methods have been successfully exploited to optimize the geometry of molecules, calculate their charge distribution, and predict or interpret their reactivity or spectroscopic features. When intermolecular interactions are being investigated, quantum mechanics (QM) modeling at the molecular mechanics (MM) level may not be adequate, and DFT methods [49] are better suited for this purpose. In fact, DFT calculations take into account electron correlation, which is required when non-covalent interactions, such as hydrogen bonds, are involved [50–52]. In DFT, several “functionals”, i.e. functions of another function, have been proposed to describe the relationship between the electron density and the energy of the molecular system under study. Here, we adopted the mPW1PW [53] functional that has been used successfully in interpreting structural and spectroscopic data for a large variety of compounds [54–57]. Our aim was to use the QM approach to investigate the interactions between L-Arg and PROP. Therefore, PROP, the protonated form of L-arginine (L-ArgH^+^), and the interacting system PROP·L-ArgH^+^, were investigated separately. A pVDZ basis set [58] and the 6–311++G** basis set have been used to describe the electronic shells of the atomic species. Natural atomic charges [59] were calculated at the optimized geometries of the same theoretical model. Solvation calculations were also carried out in the presence of water, taking into account by the Polarizable Continuum Model in its Integral Equation Formalism variant (IEF-PCM) [60]. The programs Gaussview 5.0 [61] and Molden 5.0 [62] were used to investigate the charge distributions and molecular orbital shapes. All calculations were performed with the Gaussian 09 suite of programs (rev. A.02) [63] on a E4 work station equipped with four quad-core AMD Opteron processors and 16 Gb of RAM and running the 64 bit version of the Ubuntu 12.04 Linux operating system.

### PROP responsiveness assessments after supplementation with increasing concentrations of L-Arg

Four concentrations (0.8 mM, 1.6 mM, 3.2 mM and 5.2 mM) of L-Arg (hydrochloride salt, Sigma-Aldrich, Milan, Italy) which produced increasing variations in the chemical shift in^1^H-NMR Spectroscopy experiments were added to a 3.2 mM PROP solution. For each L-Arg concentration, the measured pH value corresponded to: 7.005 ± 0.01 for PROP solution (without L-Arg); 7.01 ± 0.01 for PROP + L-Arg (0.8 mM) solution; 7.04 ± 0.01for PROP + L-Arg (1.6 mM) solution; 7.015 ± 0.01 for PROP + L-Arg (3.2 mM) solution; 7.015 ± 0.01 for PROP + L-Arg (5.2 mM) solution.

In a third session, the effect of oral supplementation with increasing concentrations of L-Arg on PROP responsiveness was assessed in each subject. The intensity of PROP bitterness and the lag time between stimulus application and the onset of bitter sensation (latency) were measured before and after oral supplementation of L-Arg. After rinsing mouth with spring water, all subjects were presented, in a random order, with 6 cups (4 mL) containing: one only PROP (3.2 mM), four PROP supplemented with L-Arg at different concentration and one only L-Arg (5.2 mM), as a control. They were instructed to swish the entire content of each cup in their mouth until they perceived the taste, to a maximum time of about 2 minutes, then to spit it out. The promptness with which bitter sensation was evoked (latency) was measured with a timer (Cole-Parmer four-channel, jumbo display clock/timer, EW-08649-10) that was started when subjects began swishing the solution and was stopped when they commenced perceiving the bitter sensation. Each stimulation was followed by oral rinsing with spring water. The interstimulus interval was set at 20 min.

The effect of oral supplementation with L-Arg on bitterness intensity of caffeine was assessed in a separate group of 45 subjects. All subjects were tested, in a random order, with 3 cups (4 mL) containing: 1.5 mM of caffeine, 6.7 mM of caffeine, and 1.5 mM of caffeine supplemented with L-Arg (1:1 caffeine:L-Arg molar ratio), respectively.

### Statistical analyses

Fisher’s method (Genopop software version 4.0; http://Kimura.univmontp2fr/~rousset/Genepop.htm) [64] was used to test *TAS2R38* and gustin gene genotype distribution and allele frequencies according to PROP status.

Basal level (unstimulated saliva) differences of the salivary L-Arg and Ps-1 according to the PROP taster status of individuals were evaluated by one-way analysis of variance (ANOVA). One-way ANOVA compared differences of the PROP bitterness intensity and latency in super-tasters, medium tasters and non-tasters and in subjects with PAV/PAV, PAV/AVI and AVI/AVI genotype of *TAS2R38*. Repeated measures ANOVA was used to compare the effect of oral supplementation with increasing concentrations of L-Arg on PROP bitterness intensity and latency in super-tasters, medium tasters and non-tasters and in subjects with PAV/PAV, PAV/AVI and AVI/AVI genotype of *TAS2R38*. Repeated measures ANOVA was also used to compare differences in bitterness intensity for two concentrations of caffeine, and the effect of oral supplementation with L-Arg on caffeine bitterness intensity. Post-hoc comparisons were conducted with the Newman-Keuls test.

Stepwise multiple linear regression was used to predict PROP bitterness intensity using *TAS2R38* and gustin genotypes, salivary L-Arg, gender, and age as predictor variables. The relative contribution of each significant variable and semipartial correlations (sr) for each variable are reported in the tables. Statistical analyses were conducted using STATISTICA for WINDOWS (version 7; StatSoft Inc, Tulsa, OK, USA). *p-*values<0.05 were considered significant.

## Results

### PROP taster status and genotyping

Molecular analysis of the *TAS2R38* locus revealed that 10 subjects were PAV homozygous, 23 were heterozygous, and 18 were AVI homozygous. Genotyping for the *rs2274333* (A/G) gustin gene polymorphism showed that 32 subjects were homozygous AA, 15 were heterozygous and only 4 were homozygous GG. Genotype distributions and allele frequencies for polymorphisms of *TAS2R38* according to PROP taster status are shown in Table 1. PROP taster groups differed statistically on the basis of their genotype distribution and allelic frequency (*χ* ^*2*^> 50.00; *p*<1.00e-008; Fisher’s test). Pairwise comparisons discriminated all groups from each other on the basis of their genotype distribution (*χ* ^*2*^>9.971; *p*≤0.0423; Fisher’s test), whereas on the basis of allelic frequency discriminated only non-tasters from the other groups (*χ* ^*2*^>50.00; *p*<1.00e-008; Fisher’s test). PROP super-taster subjects had a high frequency of PAV/PAV and PAV/AVI diplotypes, (58.33% and 41.67% respectively), and PAV haplotype (79.17%), whereas non-tasters had a very high frequency of AVI/AVI diplotype (94.74%) and AVI haplotype (97.37%).

**Table 1:** Genotype distribution and haplotype frequencies of *TAS2R38* SNPs according to PROP taster status.

	PROP status						*p*-value^a^
	super-taster		medium taster		non-tasters		
*Genotype*	*n*	*%*	*n*	*%*	*n*	*%*	
PAV/PAV	7	58.33	3	15	0	0	
AVI/AVI	0	0	0	0	18	94.74	< 0.0001
PAV/AVI	5	41.67	17	85	1	5.26	
*Haplotype*							
PAV	19	79.17	23	57.5	1	2.63	< 0.0001
AVI	5	20.83	17	42.5	37	97.37	

^a^ *p*-value derived from Fisher’s method. *n* = 51

No differences based on the genotype distribution or the allele frequencies related to the gustin gene were found (*p*>0.05; Fisher’s test) (data not shown).

### PROP taster status and salivary levels of L-Arg

**Fig 1:**
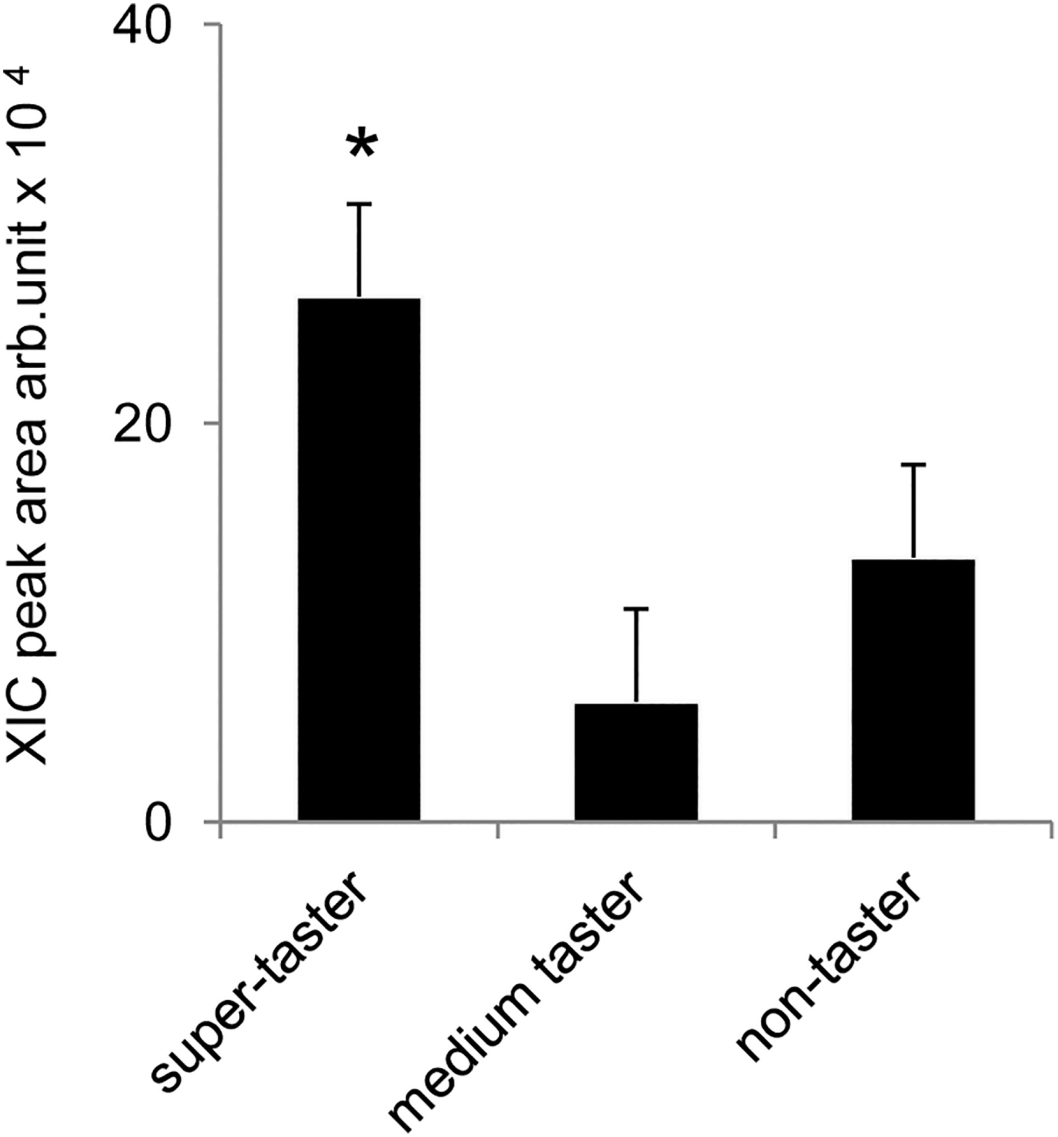
Relative concentration of free L-Arg in the PROP taster groups in unstimulated (resting) saliva. Mean values ± SEM of the extract ion current (XIC) peak areas of L-Arg amino acid determined by HPLC-ESI-IT-MS analysis. n = 51. *Significant difference from the other values (*p*≤0.024; Newman-Keuls test).

Fig 1 shows mean values ± SEM of the peak area of the ion at 60.1± 0.1 m/z determined by HPLC-ESI-MS/MS-SRM and used to assess the relative concentrations of L-Arg in unstimulated saliva of PROP super-tasters, medium tasters and non-tasters. One-way ANOVA showed that the relative concentration of L-Arg varied with the PROP taster status of subjects (*F*_[2,48]_ = 6.1228; *p* = 0.00471), being significantly higher in saliva of super-tasters than in medium tasters and non-tasters, as evidenced by post-hoc comparisons (*p*≤0.024; Newman-Keuls test). The XIC peak areas for Ps-1 protein were slightly lower in non-tasters, although the difference was not statistically significant, with respect to super-tasters and medium tasters (*F*_[2,48]_ = 0.10601; *p* = 0.8996) (see S1 Fig).

### Regression modeling to predict PROP tasting

Multiple linear regression was used to assess the relative contributions of salivary L-Arg, *TAS2R38* and gustin polymorphisms, age and gender to PROP bitterness intensity (Table 2). *TAS2R38* genotypes, L-Arg and gustin genotypes were significant predictors of PROP bitterness, with each factor contributing 53.79%, 8.07% and 4.01% to the model. The overall model predicted 67.42% of the variance in PROP bitterness intensity.

**Table 2:** Multiple regression model for PROP Bitterness (3.2 mM).

	Variable	Overall model		Parameter estimate		Each step
		(R^2^)	(*P*)	(*sr*)	(*P*)	(R^2^)
PROP bitterness	*TAS2R38*	0.6742	<0.001	0.73	<0.001	0.5379
	L-Arg			0.28	0.006	0.6185
	Gustin			0.20	0.038	0.6586
	Age			0.12	0.1866	0.6742

Independent variables included: *TAS2R38* genotypes, gustin genotypes, salivary free L-Arg, age and gender. Gender did not enter in the regression model. sr = semipartial correlation.

### ^1^H-NMR Spectroscopy-PROP/L-Arg binding

**Fig 2:**
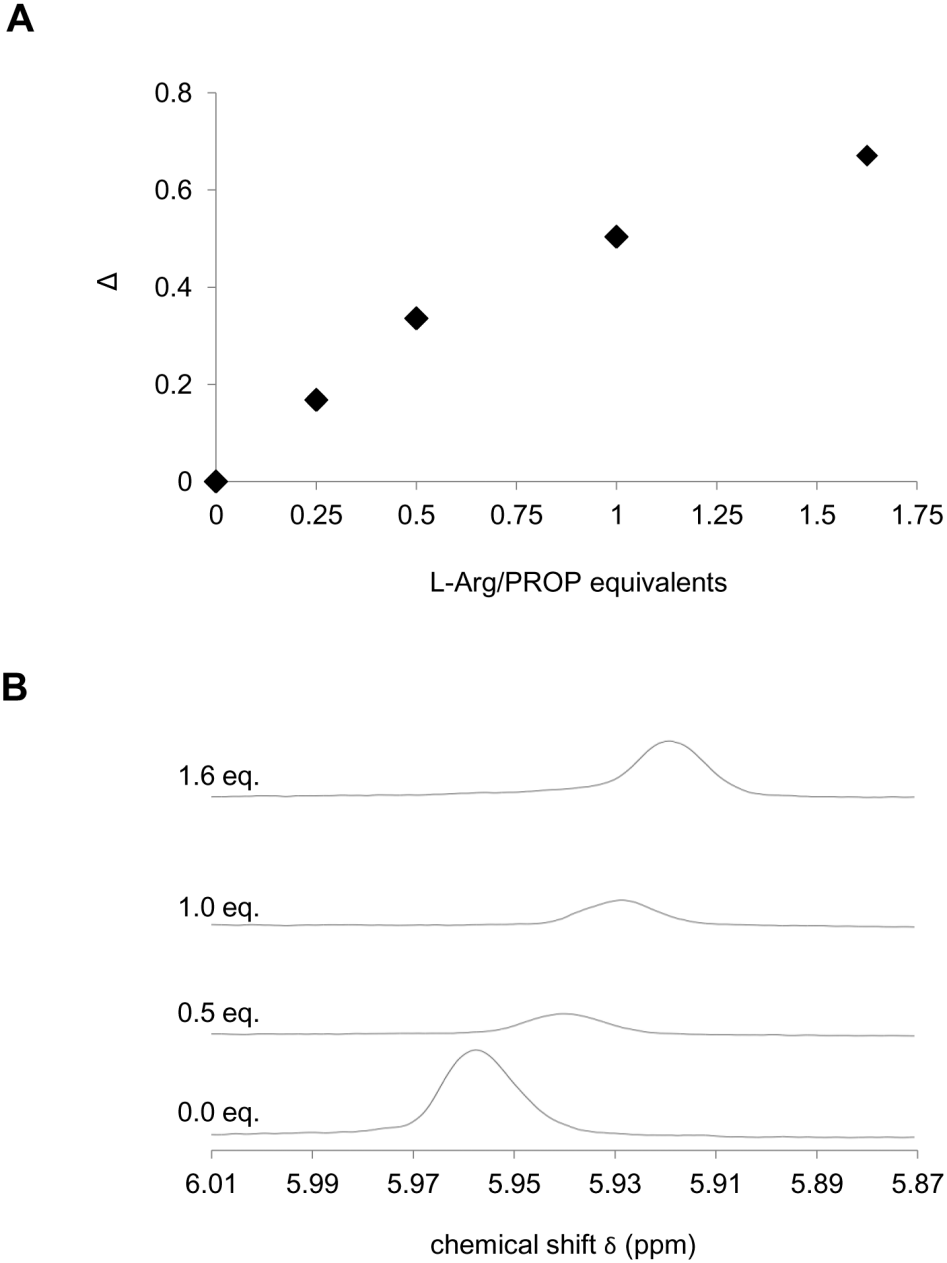
^1^H-NMR chemical shift variation supporting the formation of the PROP-L-Arg complex. PROP ring ^1^H-NMR chemical shift (δ) variation with addition of increasing amounts of L-Arg (A), and ^1^H-NMR spectra recorded on D_2_O solutions of PROP (0.005 M) upon addition of L-Arg (0.034 M) in D_2_O (B). Δ = (|(δ'-δ_0_)|/δ_0_)•100 represents the absolute value of the difference between the ^1^H-NMR signal (ppm) of the PROP ring proton in the absence (δ_0_) and in the presence (δ') of the amino acid at the relevant molar ratio, normalized for δ_0_ and expressed as a percentage. In B, the peak is the signal attributed to the PROP ring proton.

^1^H-NMR spectroscopy allowed us to determine the PROP ring proton ^1^H-NMR chemical shift variation, reported as Δ, upon the addition of increasing amounts of amino-acid up to a L-Arg:PROP molar ratio of 1.625:1 (Fig 2A and 2B). The data showed that increasing concentrations of L-Arg induced increased variations of the PROP proton field-shift and of the corresponding ^1^H-NMR signal.

### PROP/L-Arg binding computational analysis

**Fig 3:**
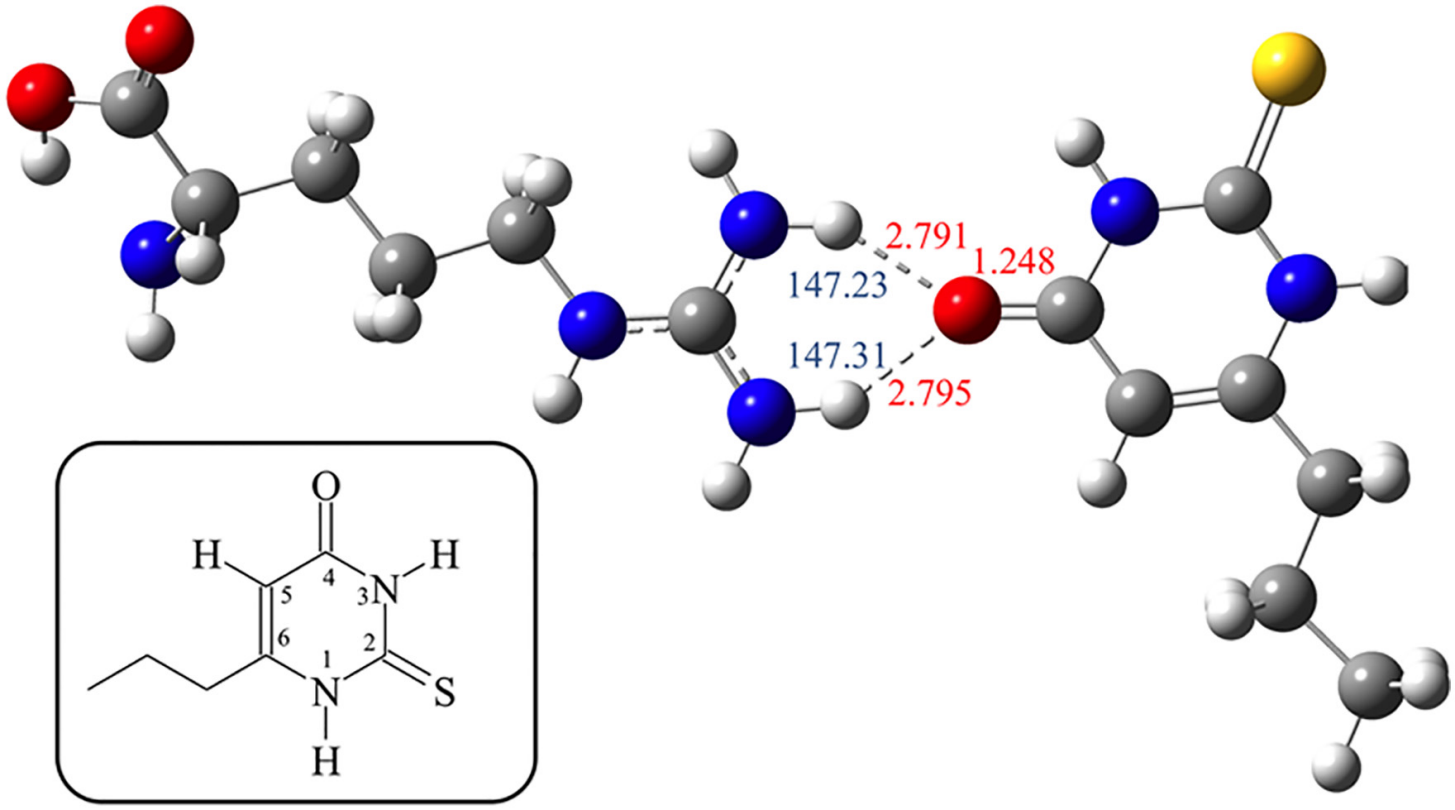
DFT Optimized structure of H-bonded adduct between PROP (right) and ArgH+ (left) calculated in water. Selected relevant interatomic distances are shown in red ink. N—H···O angles are shown in blue ink. Oxygen atoms are depicted in red, nitrogen atoms in blue, the sulfur atom in yellow, carbon in grey, and hydrogens in white. In the insert: atom numbering scheme of the heterocyclic skeleton in PROP.

Theoretical calculations at the DFT level allowed us to optimize the geometry of PROP and the protonated form of L-Arg (L-ArgH^+^). A natural population analysis [59] shows that the most negatively charged atoms of PROP are the oxygen (-0.599 and -0.598 e, with the pVDZ and 6–311++G** BSs, respectively) and the exocyclic S-atom (-0.199 and -0.185 e, with the pVDZ and 6–311++G** BSs, respectively). The interaction of the couple L-ArgH^+^/PROP to give PROP·L-ArgH^+^ was then evaluated in water by starting from different initial geometries so to achieve both the O-bonded and the S-bonded isomers. In both cases, the—NH_2_ terminal group of L-ArgH^+^ interacts with the carbonyl or thiocarbonyl groups of PROP by forming two hydrogen bonds (average H···O = C distance 2.793 Å; N-H···O angle 147.27°) (Fig 3). The interaction energies between the two synthons are 4.04 and 6.49 kcal mol^–1^ for the system interacting through the C = S and C = O groups, respectively, with the 6–311++G** BS. Natural charges calculated on the systems PROP and PROP·L-ArgH^+^ provided the charge distribution (C atom in position 5: PROP, –0.371 e; PROP·L-ArgH^+^ = –0.363 and –0.314 e. N atom in position 1: PROP, –0.570; PROP·L-ArgH^+^ = –0.565 and –0.563 e. N atom in position 3: PROP, –0.603; PROP·L-ArgH^+^ = –0.593 and –0.597 e depending on weather the interaction with L-ArgH^+^ involves the C = S and C = O groups, respectively; see inset in Fig 3 for the numbering scheme).

### Effect of oral supplementation of L-Arg on PROP responsiveness

**Fig 4:**
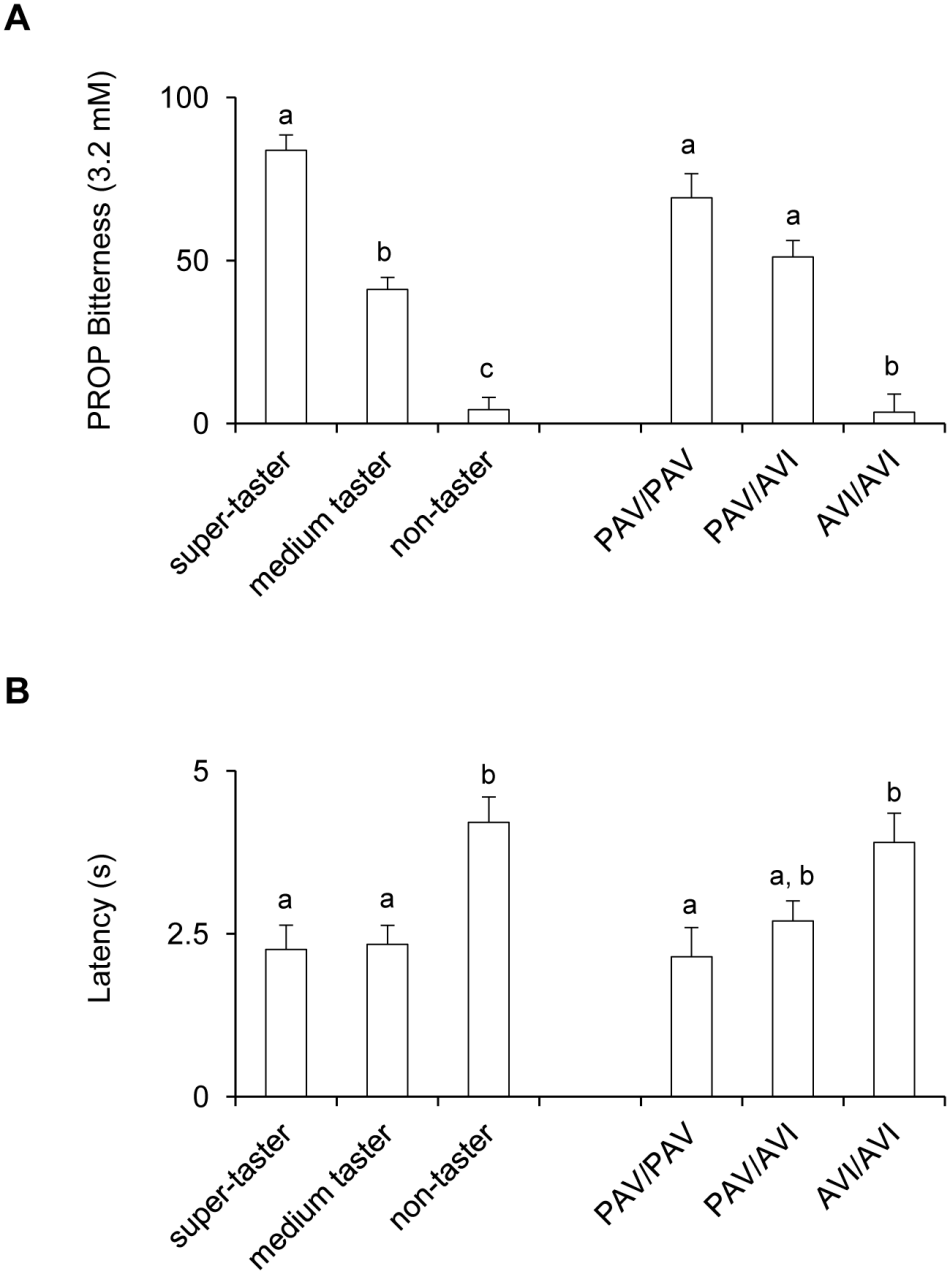
PROP responsiveness in subjects phenotyped for PROP tasting and genotyped for *TAS2R38*. Bitterness intensity ratings (A) and latency (B) for a 3.2 mM PROP solution in super-tasters, medium tasters and non-tasters and in individuals with *TAS2R38* genotypes PAV/PAV, PAV/AVI and AVI/AVI. All values are mean (± SEM). n = 51. Different letters indicate significant differences (*p*<0.0235; Newman-Keuls test).

Fig 4 shows bitterness intensity ratings (A) and latency (B) for 3.2 mM PROP solution in super-tasters, medium tasters and non-tasters and in individuals with *TAS2R38* genotypes PAV/PAV, PAV/AVI and AVI/AVI. One-way ANOVA showed that PROP bitterness intensity and latency varied with taster status and with *TAS2R38* genotypes (bitterness: *F*_[2,48]_>31.392; *p*<0.00001; latency: *F*_[2,40]_>4.0879; *p*<0.0246). Post-hoc comparisons showed that bitterness intensity ratings were statistically higher in super-tasters than in medium tasters, and medium tasters gave higher intensity ratings to PROP than non-tasters (*p* = 0.000126; Newman-Keuls test). Latency values were statistically higher in non-tasters than in the other groups (*p*<0.0026; Newman-Keuls test), but no difference between medium and super-tasters was found (*p*>0.05; Newman-Keuls test). In addition, pairwise comparisons showed lower bitterness intensity ratings in individuals with genotype AVI/AVI than in those with the other genotypes (*p* = 0.000128; Newman-Keuls test), but no differences between PAV/PAV and PAV/AVI individuals (*p* = 0.12). Latency was statistically higher in individuals with the AVI/AVI genotype than in those with the PAV/PAV genotype (*p* = 0.0235; Newman-Keuls test). No latency differences were found for heterozygous individuals relative to the other genotypes (*p*>0.08).

**Fig 5:**
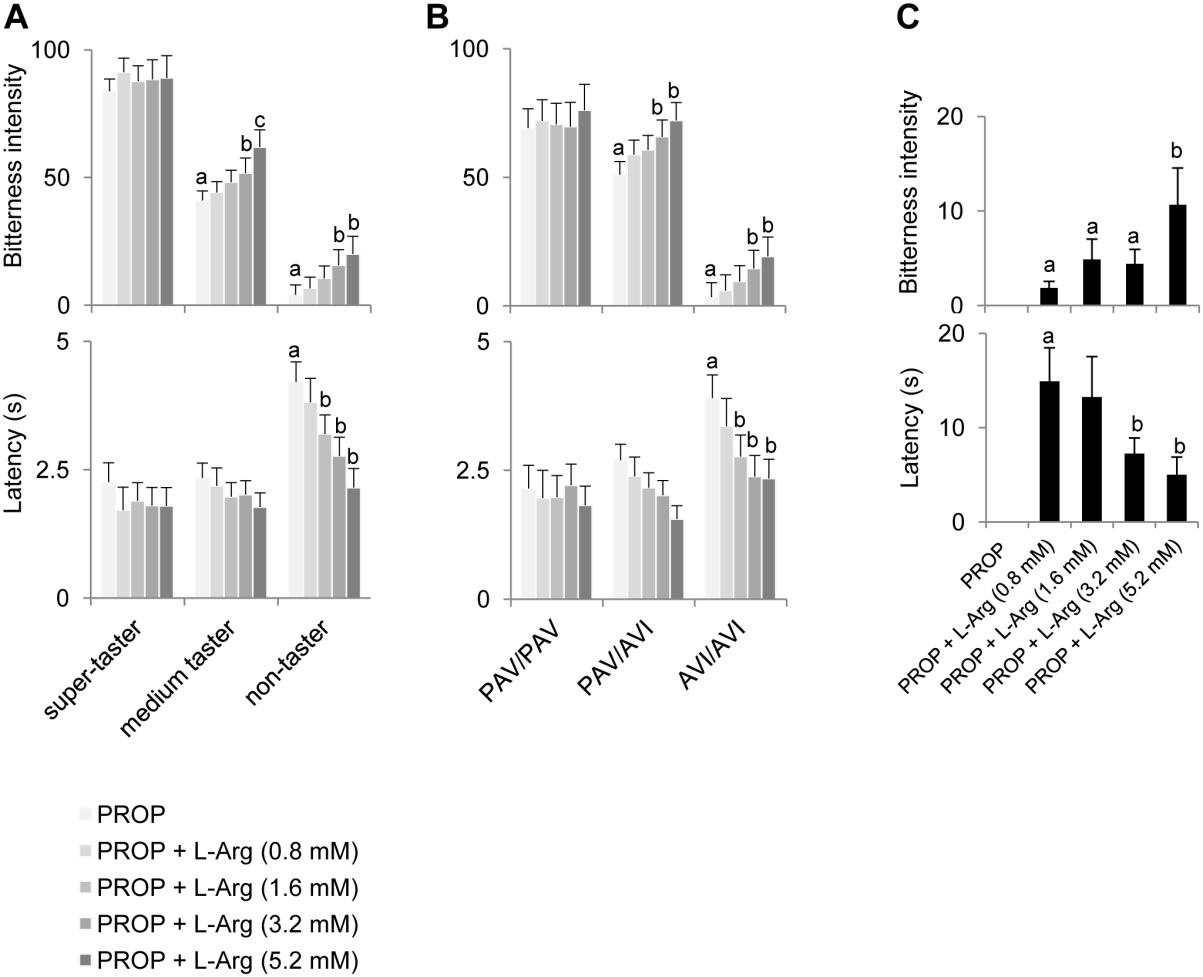
Effect of L-Arg supplementation on PROP responsiveness. Mean values ± SEM of bitterness intensity ratings and latency evoked by a 3.2 mM PROP solution and the same PROP solution (3.2 mM) supplemented with increasing concentrations of L-Arg (0.8, 1.6, 3.2 and 5.2 mM) in super-tasters, medium tasters and non-tasters (n = 51) (A); or in individuals with genotypes PAV/PAV, PAV/AVI and AVI/AVI of *TAS2R38* (n = 51) (B). A sub-set of non-taster subjects (n = 8) who do not perceive 3.2 mM PROP is shown in C. Different letters indicate significant differences (*p*<0.049; Newman-Keuls test subsequent to repeated measures ANOVA).

Fig 5A shows the effects of oral supplementation with L-Arg on PROP responsiveness as a function of PROP taster group. Supplementation with 3.2 mM L-Arg (1:1 PROP:L-Arg molar ratio) increased PROP bitterness intensity (upper graph) in medium tasters and non-tasters (*p*<0.031; Newman-Keuls test subsequent to repeated measures ANOVA). However, at the highest L-Arg concentration (5.2 mM, 1:1.625 molar ratio) only medium tasters experienced a further increase in bitterness intensity (*p* = 0.00002; Newman-Keuls test subsequent to repeated measures ANOVA). No changes in PROP bitterness intensity were found in super-tasters (*p*>0.05). The effect of L-Arg on latency (lower graph) was restricted to non-taster subjects. Supplementation with L-Arg at 1.6 mM (1:0.5 molar ratio) and at higher concentrations decreased latency in these subjects (*p* = 0.025; Newman-Keuls test subsequent to repeated measures ANOVA). No latency changes were found in super-tasters and medium tasters.

Fig 5B shows the same data as a function of *TAS2R38* genotypes. The effects of L-Arg on PROP bitterness intensity (upper graph) was restricted to heterozygous and homozygous AVI/AVI subjects. Supplementation with 3.2 mM and 5.2 mM L-Arg significantly increased bitterness intensity in these subjects (*p*<0.0035; Newman-Keuls test subsequent to repeated measures ANOVA). No changes in PROP bitterness intensity were found in PAV/PAV subjects. The effects of L-Arg on latency (lower graph) were restricted to AVI/AVI subjects, and was apparent beginning at 1.6 mM L-Arg and at higher concentrations (*p* = 0.015; Newman-Keuls test subsequent to repeated measures ANOVA). No changes in latency were found in PAV/PAV and PAV/AVI subjects.

We identified eight subjects in the non-taster group who perceived no bitterness from the 3.2 mM PROP solution without added L-Arg. After supplementation with L-Arg (Fig 5C **)**, these individuals experienced PROP bitterness for the first time. In particular, six of them perceived PROP bitterness when supplemented with L-Arg at the lowest concentration tested (0.8 mM), whereas all of these subjects perceived PROP bitterness at the highest concentration (5.2 mM) Repeated measures ANOVA showed that bitterness intensity ratings increased and latency decreased as a function of L-Arg concentration (bitterness: *F*_[3,21]_ = 6.798; *p* = 0.0022; latency: *F*_[3,15]_ = 4.666; *p* = 0.017).

Bitterness intensity ratings and latency for a 3.2 mM PROP solution and 3.2 mM PROP solutions supplemented with increasing concentrations of L-Arg according to gustin gene *rs2274333* (A/G) polymorphism is shown in S2 Fig. Bitterness intensity ratings of individuals with the GG genotype were lower by more than 20% compared to those with AA and AG genotypes. However these differences were not statistically significant due to an inadequate number of subjects (4) having this genotype in the sample. The effect of L-Arg supplementation was restricted to homozygous GG subjects in whom supplementation with only the highest L-Arg concentration (5.2 mM) was effective in increasing bitterness intensity (*p* = 0.004; Newman-Keuls test subsequent to repeated measures ANOVA). No other changes related to gustin genotypes were found.

**Fig 6:**
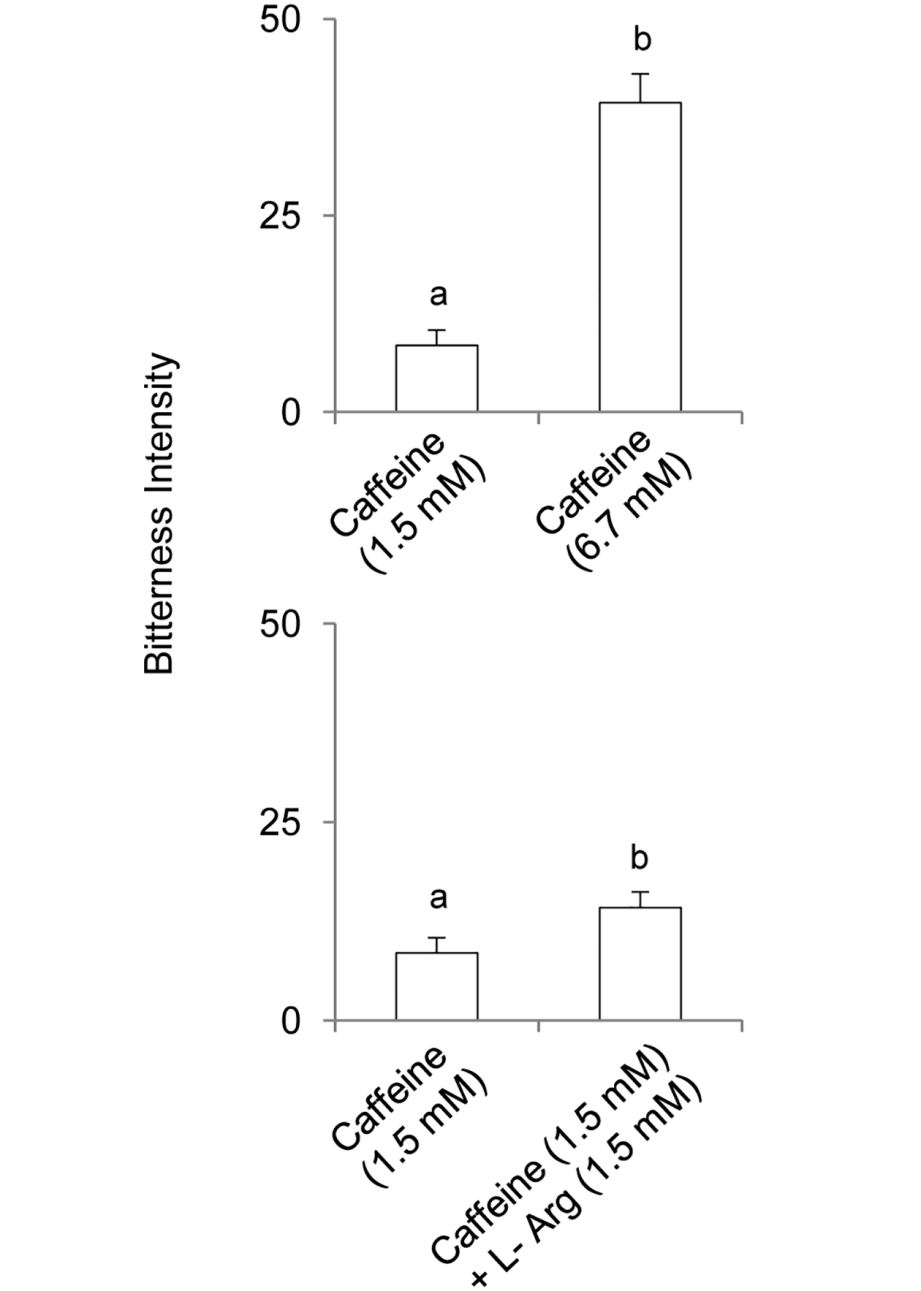
Effect of L-Arg supplementation on bitterness intensity of caffeine. Bitterness intensity ratings for 1.5 and 6.7 mM of caffeine (upper graph), and bitterness intensity ratings for 1.5 of caffeine and 1.5 mM caffeine supplemented with L-Arg (1:1 caffeine:L-Arg molar ratio) (lower graph). Different letters indicate significant differences (*F*_[1,40]_ = 15.625; *p* = 0.00031; repeated measures ANOVA).

Bitterness intensity ratings for 1.5 and 6.7 mM caffeine (upper graph) and the effect of oral supplementation with 1.5 L-Arg on bitterness intensity of caffeine (lower graph) are shown in Fig 6. Bitterness intensity ratings for 1.5 mM caffeine were lower than those for 6.7 mM caffeine (*F*_[1,40]_ = 87.266; *p*<0.00001; repeated measures ANOVA) (upper graph in Fig 6). Supplementation with L-Arg (1:1 caffeine:L-Arg molar ratio) significantly increased caffeine bitterness intensity (*F*_[1,40]_ = 15.625; *p* = 0.00031; repeated measures ANOVA) (lower graph in Fig 6).

All solutions containing only L-Arg did not evoke any taste perception in all subjects (data not shown).

## Discussion

The role of various salivary components in taste perception has been long recognized [27, 35, 36, 65–69]. In a previous study, were reported that specific salivary peptides belonging to the basic proline-rich protein family (bPRP) and their constituent amino acids were important factors contributing to PROP taste perception. Data showed that the highest levels of Ps-1 protein in saliva were associated with the highest responsiveness to PROP [35, 36], and that the oral supplementation with Ps-1 in individuals lacking this protein in saliva (non-tasters) enhanced PROP responsiveness [36]. Furthermore, oral supplementation with the free form of L-Arg, a constituent amino acid of Ps-1 protein, increased PROP bitterness intensity in both medium tasters and non-tasters [36]. In addition, ^1^H-NMR experiments showed that only L-Arg and L-Lys, among all the amino acids in the Ps-1 sequence, chemically interact with the PROP molecule, and the interaction involving L-Arg is stronger than that involving L-Lys [36]. Since L-Lys and L-Arg are the only amino acids with amino groups in the side chain among those studied, ^1^H-NMR results suggest that the chemical interaction could involve these amino groups and the carbonyl/thiocarbonyl groups of the PROP heterocycle. In parallel, psychophysical data strongly support the ^1^H-NMR results, showing that L-Arg enhances PROP bitterness more than L-Lys.

The present results confirm and extend these initial findings, demonstrating that salivary levels of free L-Arg contribute to PROP phenotypic differences. Specifically, we showed that the highest levels of free L-Arg in resting saliva are associated with the highest responsiveness to PROP (super-tasters), and that oral supplementation with L-Arg in individuals with lower salivary levels of this amino acid (medium tasters and non-tasters) enhances PROP responsiveness in a dose-dependent manner. These effects were related to both PROP taster phenotypes and, *TAS2R38* genotypes. Importantly, the enhancement in PROP bitterness intensity was restricted to medium tasters and non-tasters or heterozygous and homozygous AVI/AVI subjects in whom the L-Arg supplementation was effective in a molar ratio of 1:1 with the PROP molecule. The fact that L-Arg alone was tasteless at the concentrations we used rules out the possibility that L-Arg contributed to bitter taste enhancement on its own. Interestingly, our previous findings [36] showed that oral supplementation with L-Arg but not Ps-1 protein increased PROP bitterness in medium tasters. This result implies that L-Arg enhances PROP bitterness in medium tasters who already have high levels of the Ps-1 protein in saliva, and giving additional Ps-1 leads to no further enhancement of this effect.

We observed only a small effect of L-Arg supplementation on PROP bitterness related to gustin genotypes probably due to the low frequency of the G allele in our sample. According to regression modeling, *TAS2R38* genotype was the major predictor of PROP bitterness intensity followed by salivary L-Arg. However, gustin polymorphisms were also significant predictors in the model.

It has been known for a long time that bitter taste intensity grows more slowly over time compared to other taste qualities, and it tends to persist over time [70–72]. To our knowledge, these features of PROP taste responsiveness have not been studied. In the present experiments, we measured the latency (lag time) between stimulus application and onset of PROP bitterness. We observed short latencies (~2.5 s) in super-tasters and medium tasters. However, the latency to perceive PROP bitterness was almost twice as high (~5.0 s) in non-tasters compared to the other groups. Supplementation with L-Arg decreased bitterness latency in a dose-dependent manner in non-tasters or AVI/AVI subjects and this effect was observed beginning at the 1.6 mM concentration. Furthermore, at the highest L-Arg concentration, latency in non-tasters matched the latencies in the other groups. The ineffectiveness of L-Arg supplementation in reducing latency in medium tasters or PAV/AVI subjects could be due to a high concentration of Ps-1 in saliva of individuals in this group, who already showed a short latency when they tasted PROP solution without L-Arg. It is worth noting that most (85%) of medium tasters had at least one AVI haplotype of *TAS2R38* and almost all (95%) non-tasters were homozygous for the AVI haplotype.

We also found that a sub group of non-tasters who experienced no bitterness from PROP alone, were able to taste PROP for the first time when it was supplemented with L-Arg, and increasing concentrations of L-Arg resulted in greater PROP bitterness intensity and shorter latencies in these individuals. Importantly, all of these individuals carried the AVI/AVI diplotype. Thus, L-Arg supplementation may permit activation of the TAS2R38 receptor in individuals who exclusively express the AVI, so-called ‘nonfunctional’ form of the receptor.

As a whole, these results suggest that the L-Arg facilitates PROP perception, even when subjects have the specific receptor nonfunctional (AVI) form, by causing a significant increase of PROP bitterness intensity in subjects who had low salivary levels of L-Arg (medium taster and non-tasters), and a reduction of latency in subjects with low levels of both L-Arg and of Ps-1 protein (non tasters).

During granule maturation many salivary proteins are cleaved at the level of arginine residues by the action of convertases, followed by removal of the C-terminal arginine residues by specific carboxypeptidases [73]. C-terminal removal also takes place after secretion into the mouth [73]. Since we found that the highest responsiveness to PROP (super-tasters) is associated with high levels of L-Arg in saliva, these findings could suggest that the activity of these enzymes (convertases and carboxypeptidases) may also play a role in modulating the expression of the PROP phenotype. Future studies will examine this possibility.

To better understand the physiological mechanism by which L-Arg facilitates PROP bitterness intensity, we used ^1^H-NMR spectroscopy to investigate the chemical interaction between PROP and increasing amounts of free L-Arg. These results showed that the addition of L-Arg to PROP solution up to a molar ratio of 1.625:1, induced increases in the PROP proton field-shift variation (Fig 2), an indicator of the chemical interaction strength between the two molecules.

DFT calculations shed additional light on the nature of the interaction between L-Arg and PROP. L-Arg is a basic amino acid (pK_r_ = 12.48) that remains protonated at the guanidine residue in the free form and in proteins [74, 75], and lipid membranes [76], across the entire physiological pH range. In our computations, only the terminal guanidinium unit of the side chain of L-ArgH^+^ is available for interaction with the PROP molecule. The metric parameters optimized for PROP are in very good agreement with the structural data deposited at the Cambridge Crystallographic Data Centre [77]. The calculated charge distribution on the isolated PROP molecule indicates that both the C = O and C = S groups can behave as hydrogen-bond acceptors. A structural investigation led to the conclusion that the hydrogen-bonding capability of carbonyl and thiocarbonyl groups seem to be comparable [77]. Therefore, the interaction of the couple L-ArgH^+^/PROP was evaluated by considering two isomers, featuring L-ArgH^+^ interacting with either the C = O or C = S groups of PROP. In both cases, calculations showed that the—NH_2_ group of L-ArgH^+^ side chain interacts with the carbonyl or thiocarbonyl groups of PROP by forming two hydrogen bonds. An examination of the relative stabilization energies calculated for PROP·L-ArgH^+^ in water clearly indicates that the interaction of ArgH^+^ with the C = O group of PROP is only slightly favored relative to the interaction involving the C = S group. Therefore, at room temperature both interactions are likely to occur. The interaction with L-ArgH^+^ slightly modifies the charge distribution on the PROP unit, possibly determining the modest variations in the ^1^H-NMR chemical shift discussed above.

The interaction of PROP with the TAS2R38 receptor has been recently studied [78–80], and would likely occur through the N—H [80] or the C = S/C = O [79] groups of PROP. In particular, the N atom in position 1 (inset in Fig 3) may be involved in the binding of the AVI receptor form while the N atom in position 3 may be involved in the binding of the PAV form [80]. MM/CG calculations by Marchiori et al. 2013 showed that the C = S group is involved in the interaction with the receptor through H-bonds [79]. Notably, the H-bond formation of PROP·L-ArgH^+^ results in the lowering of the charges calculated on the N-atoms of PROP. This reduction in charge implies that the N—H groups of PROP are not stearically hindered as would be the case for free PROP (Fig 3), but are possibly ‘activated’ for further interactions with receptor binding sites.

Based on these findings we propose the following mechanism to describe the permissive role of L-Arg in PROP perception. The protonated residue of L-Arg both as a free amino acid and as a constituent of the salivary Ps-1 protein can interact with the C = O or C = S groups of PROP by forming two hydrogen bonds. In principle, the PROP molecule could interact with the terminal guanidinium group of L-Arg, but also with its carboxylate moiety, thus making the L-Arg-PROP complex more available to activate the specific receptor site than PROP alone. Therefore, L-Arg and/or Ps-1 can be viewed as ‘carriers’ of the PROP molecule in saliva, possibly increasing its solubility in aqueous media due to the formation of the charged adduct. Since other free amino acids, such as L-Lys and L-His, that display a terminal amino-group have been found in saliva [81], we cannot exclude that they could chemically interact with the PROP molecule and show similar effects. However, the double hydrogen bond that only L-Arg can form could be responsible for the greater effect of this amino acid.

The notion that salivary proteins solubilize taste molecules to enhance receptor binding has been previously suggested [82]. The formation of C = O···H-N and/or C = S···H-N hydrogen bonds between PROP and/or these potential carriers may make this complex more available for interacting with receptor sites than free PROP, thus enhancing PROP responsiveness. The viability of this potential mechanism needs to be empirically supported with receptor binding studies. However, we also observed that supplementation with L-Arg had a similar effect on bitterness intensity of caffeine, which is detected by five different TAS2Rs [83]. Together, these data point to a role for L-Arg in facilitating bitterness intensity of the tastant probably modifying the solubility of these molecules, to enhance their availability at receptor sites, rather than an effect of L-Arg on binding of these tastants with their specific receptors. These results suggest that L-Arg has the potential to enhance the bitterness of various bitter substances. This possibility should be examined in future experiments.

In conclusion, it is becoming increasingly clear that PROP tasting is a complex phenotype influenced by a variety of factors including the functional interaction between *TAS2R38* and the gustin gene that controls the taste bud trophic factor known as gustin protein [28], differences in messenger RNA expression in fungiform papillae [84], as well as environmental factors such as age and smoking [85, 86]. Our recent work adds an additional dimension to our understanding of these phenotypic differences by focusing on the permissive role of the salivary Ps-1 protein and its constituent amino acid L-Arg in PROP taste responsiveness [35, 36]. Here we showed in psychophysical experiments that free L-Arg is capable of enhancing PROP responsiveness in a dose-dependent manner, and in parallel with QM theoretical calculations showed that free L-Arg could facilitate the availability of PROP molecule at receptor site, with hydrogen bonding being the fundamental chemical interaction between PROP and L-Arg. Additional L-Arg appears to be most effective in enhancing PROP bitterness when salivary free L-Arg and Ps-1 levels are low, as we have observed in PROP non-tasters.

Finally, it has been shown that small peptides and amino acid derivatives mask the bitter taste of foods [87]. In particular, L-Arg and L-Lys are known to suppress the bitterness of quinine by specifically blocking the T2R4 receptor [87–89]. The observations that L-Arg has opposing effects on bitterness at different T2R receptors underscores the versatility of amino acids and other small molecules in modulating the taste system. Together, these findings suggest that oral supplementation with L-Arg could be a strategy for selectively modifying taste responses.

## Supporting Information

S1 Fig Relative concentrations of Ps-1 protein in the PROP taster groups in unstimulated (resting) saliva. Mean values ± SEM of the extract ion current (XIC) peak areas of Ps1 protein determined by HPLC-ESI-IT-MS analysis in unstimulated saliva of PROP super-tasters, medium tasters and non-tasters. n = 51.
(TIF)

S2 Fig Effect of L-Arg supplementation on PROP responsiveness according to gustin gustin *rs2274333* (A/G) polymorphism. Bitterness intensity ratings and latency for a 3.2 mM PROP solution and 3.2 mM PROP solutions supplemented with increasing concentrations of L-Arg in individuals with genotypes AA, AG and GG. All values are mean (±SEM). n = 51. Different letters indicate significant differences (*p*<0.033; Newman-Keuls test subsequent to repeated measures ANOVA).
(TIF)

S1 Table Classification of subjects by PROP taster status. Values are means ± SEM of ratings of perceived taste intensity in response to three concentrations of PROP and NaCl by PROP taster groups. n = 51. Three-way ANOVA was used to compare PROP intensity ratings with NaCl intensity ratings across groups (*F*_[4,288]_ = 17.790; *p*<0.00001). * = significant difference between PROP and the corresponding NaCl concentration (*p*<0.0001; Newman-Keuls test).
(DOCX)
