# Supplementary figures and images for: Dose-Dependent Effects of L-Arginine on PROP Bitterness Intensity and Latency and Characteristics of the Chemical Interaction between PROP and L-Arginine
Source: PLoS One. 2015 Jun 23;10(6):e0131104. doi: 10.1371/journal.pone.0131104 (PMC4477953; doi:10.1371/journal.pone.0131104)

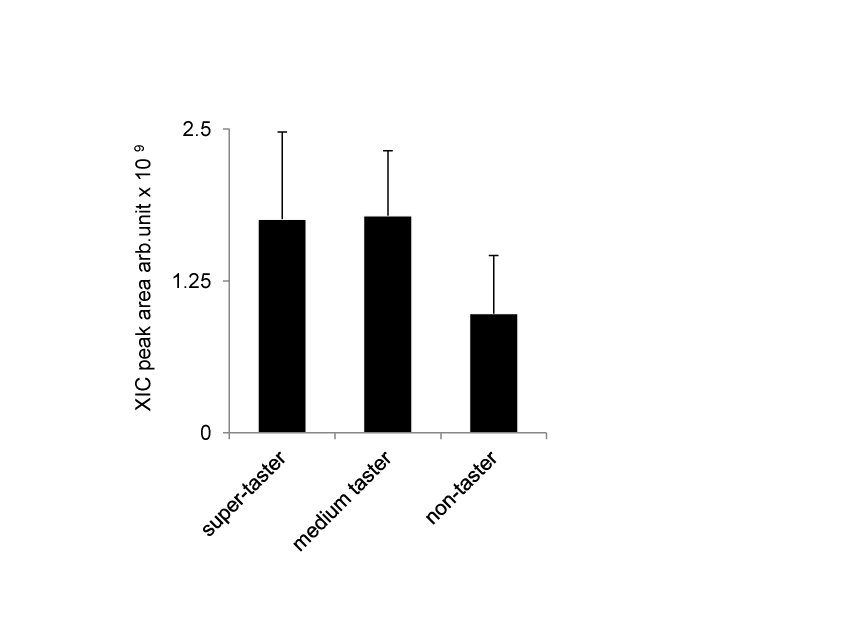

Supplement: S1 Fig — Mean values ± SEM of the extract ion current (XIC) peak areas of Ps1 protein determined by HPLC-ESI-IT-MS analysis in unstimulated saliva of PROP super-tasters, medium tasters and non-tasters. n = 51. (TIF) [file pone.0131104.s001.tif]

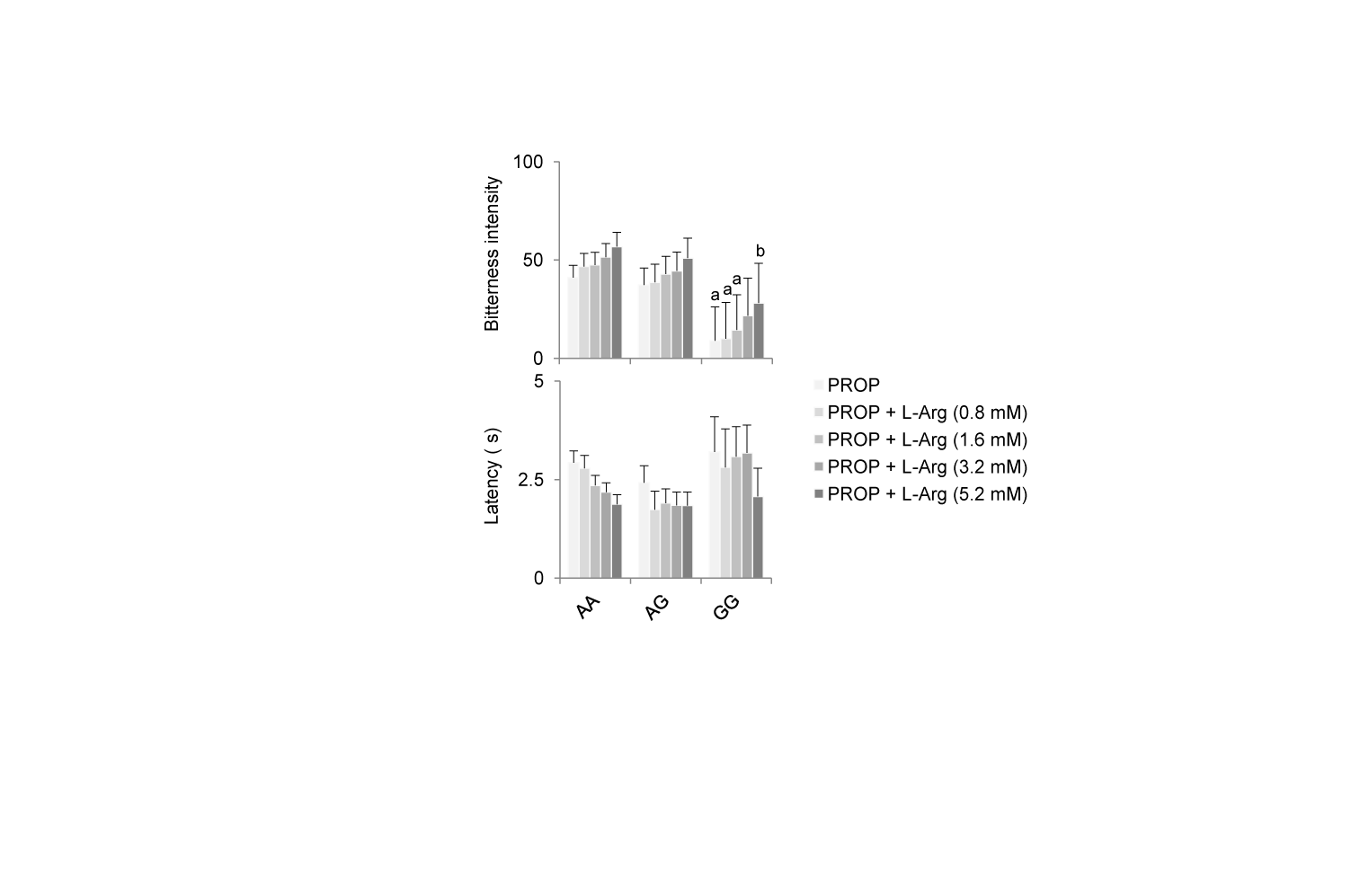

Supplement: S2 Fig — Bitterness intensity ratings and latency for a 3.2 mM PROP solution and 3.2 mM PROP solutions supplemented with increasing concentrations of L-Arg in individuals with genotypes AA, AG and GG. All values are mean (±SEM). n = 51. Different letters indicate significant differences (p<0.033; Newman-Keuls test subsequent to repeated measures ANOVA). (TIF) [file pone.0131104.s002.tif]
