# Supplementary material for: Dose-Dependent Effects of L-Arginine on PROP Bitterness Intensity and Latency and Characteristics of the Chemical Interaction between PROP and L-Arginine
Source: PLoS One. 2015 Jun 23;10(6):e0131104. doi: 10.1371/journal.pone.0131104 (PMC4477953; doi:10.1371/journal.pone.0131104)
Supplement: S1 Table — Values are means ± SEM of ratings of perceived taste intensity in response to three concentrations of PROP and NaCl by PROP taster groups. n = 51. Three-way ANOVA was used to compare PROP intensity ratings with NaCl intensity ratings across groups (F [4,288] = 17.790; p<0.00001). * = significant difference between PROP and the corresponding NaCl concentration (p<0.0001; Newman-Keuls test). (DOCX) [file pone.0131104.s003.docx]

**Table S1.**

|  | super-tasters  (*n*=12) | medium tasters  (*n*=20) | non-tasters  (*n*=19) |
| --- | --- | --- | --- |
| PROP |  |  |  |
| 0.032 mM | 6.28 ± 1.39 | 3.36 ± 0.97 | 0.65 ± 0.26 |
| 0.32 mM | 39.75 ± 3.68* | 31.65 ± 4.16 | 3.39 ± 0.89* |
| 3.2 mM | 83.22 ± 4.03* | 58.71 ± 4.53 | 21.38 ± 3.76* |
|  |  |  |  |
| NaCl |  |  |  |
| 0.01 M | 1.66 ± 0.62 | 2.22 ± 0.98 | 3.79 ± 1.37 |
| 0.1 M | 14.59 ± 2.29* | 21.47 ± 1.61 | 31.82 ± 4.18* |
| 1 M | 39.49 ± 3.35* | 58.14 ± 4.49 | 64.40 ± 4.92* |
